# Explaining the differences of gait patterns between high and low-mileage runners with machine learning

**DOI:** 10.1038/s41598-022-07054-1

**Published:** 2022-02-22

**Authors:** Datao Xu, Wenjing Quan, Huiyu Zhou, Dong Sun, Julien S. Baker, Yaodong Gu

**Affiliations:** 1 Faculty of Sports Science, Ningbo University, Ningbo, 315211 China; 2 Faculty of Engineering, University of Pannonia, Veszprém, Hungary; 3 Savaria Institute of Technology, Eötvös Loránd University, Budapest, Hungary; 4 School of Health and Life Sciences, University of the West of Scotland, Glasgow, G72 0LH Scotland, UK; 5 Department of Sport, Physical Education and Health, Hong Kong Baptist University, Hong Kong, 999077 China

**Keywords:** Bone quality and biomechanics, Risk factors, Biomedical engineering, Data processing, Machine learning

## Abstract

Running gait patterns have implications for revealing the causes of injuries between higher-mileage runners and low-mileage runners. However, there is limited research on the possible relationships between running gait patterns and weekly running mileages. In recent years, machine learning algorithms have been used for pattern recognition and classification of gait features to emphasize the uniqueness of gait patterns. However, they all have a representative problem of being a black box that often lacks the interpretability of the predicted results of the classifier. Therefore, this study was conducted using a Deep Neural Network (DNN) model and Layer-wise Relevance Propagation (LRP) technology to investigate the differences in running gait patterns between higher-mileage runners and low-mileage runners. It was found that the ankle and knee provide considerable information to recognize gait features, especially in the sagittal and transverse planes. This may be the reason why high-mileage and low-mileage runners have different injury patterns due to their different gait patterns. The early stages of stance are very important in gait pattern recognition because the pattern contains effective information related to gait. The findings of the study noted that LRP completes a feasible interpretation of the predicted results of the model, thus providing more interesting insights and more effective information for analyzing gait patterns.

## Introduction

With an increase of the number of recreational runners, the injuries caused by overuse running are increasing^1,2^. The etiology of excessive use of running injuries is multifactorial, which may result from the interaction of many factors of external uncertainties (e.g., weekly running days, weekly running mileages, running environment, footwear) and internal risk (e.g., biomechanics factors, foot strike pattern, anatomic factors, age, gender)^3^. The injury rate among recreational runners has been recorded as high as 29.4%, with overuse knee injuries (e.g., knee anterior pain and iliotibial band syndrome) being the most reported^4^. Previous studies have shown that weekly running mileage is a major risk factor related to running injuries^1,5^, and there are significant differences in injuries between higher-mileage runners (self-reported running more than 32 km per week) and low-mileage runners (self-reported running less than 25 km per week)^6^. The higher-mileage weekly runners show higher rates of hip and hamstring injuries^7^, while the low-mileage weekly runners show higher rates of knee injuries^8^. Gait patterns are an important factor in decoding gait characteristics, which is related to revealing motor injuries and gait recognition^9,10^. Therefore, running gait patterns have implications for understanding the causes of injuries between higher-mileage runners and low-mileage runners. However, there is limited research on the possible relationship between running gait patterns and weekly running mileages.

Biomechanical analysis of higher-mileage and low-mileage runners may be useful in order to better understand the potential relationship between running mileage and specific types of injuries. However, current research on the biomechanical performance of running gait of high-mileage and low-mileage runners mainly focuses on kinematics. Boyer et al. used the principal component analysis found that there were recognizable differences in the kinematics of the sagittal and frontal planes of the ankle, the frontal plane of the knee, the frontal and transverse plane of the hip in the stance phase between high-mileage and low-mileage runners^11^. Clermont et al. then combined the methods of principal component analysis with support vector machines with kinematic data to classify runners based on mileage, and found that the classification performance of gait kinematics of high-mileage and low-mileage runners had high accuracy, which means there was high identifiability in the gait kinematics between high-mileage and low-mileage runners^12^. However, the kinetics (joint moments) of biomechanical parameters also play an important role in identifying damage patterns, especially in revealing the stresses on the major joints^13,14^. Therefore, both kinematics and kinetics should be considered to improve the recognition of gait patterns and reveal the pattern characteristics in a more detailed way when recognizing the running gait patterns of high-mileage and low-mileage runners.

When analyzing variables related to gait patterns, the previous method mainly examines the influence of single-time discrete gait variables. Previous methods have successfully addressed many important clinical and scientific questions related to human gait, but there are some inherent limitations. For example, when discrete variables are extracted from time-series variables, a large amount of data is lost ^10^. In addition, a single pre-selected gait variable may miss potentially meaningful information represented by other unselected variables and correlated variables. Therefore, given the shortcomings of traditional methods, machine learning techniques (such as hierarchical clustering analysis, support vector machines, artificial neural networks, etc.) and multivariable statistical analysis have been used to examine and analyze human motion based on time-series gait patterns in recent years^9,12,15–17^. The progressive development of advanced motion capture equipment makes it possible to collect a large amount of clinical biomechanics data, which results in the increasing application of machine learning in clinical biomechanics^16,18,19^. For example, artificial neural networks and support vector machines are used for pattern recognition and classification of gait features to emphasize the uniqueness of gait patterns^9,10,12^.

Machine learning approaches can be very successful in solving many clinical biomechanical problems related to classification systems and providing new insights from complex model systems. However, they all have the same problem of being a black box that doesn't provide any information about what makes the decisions^20,21^. In other words, these models often lack the interpretability of the predicted results of the classifier^22^. The main reason for this lack of interpretability is the nonlinearity of various mappings that process the original data set (such as gait patterns) to their characteristic representation and then to the classifier function. In gait pattern recognition, this prevents experts in the relevant fields from carefully verifying classification decisions, because simple answers of "yes" or "no" sometimes have little or limited value. Therefore, Layer-wise Relevance Propagation (LRP) technology is proposed to solve the problem of lack of interpretability^22^. LRP is a technology used to identify important relevance (that is, by measuring the contribution of each input variable to the overall predict outcomes) through backward propagation in neural networks^22,23^. LRP has been successfully applied to classification recognition tasks in many scenarios, such as text, image, and even gait pattern recognition^9,10,24^. Therefore, the application of LPR in running gait pattern recognition can improve the overall transparency of the classifier and make the classification results interpretable, thus providing reliable clinical biomechanical diagnostic results.

Therefore, the purpose of this study was to investigate the differences in running gait patterns between higher-mileage runners and low-mileage runners. Specifically, the aim of this study was: (1) To train a deep neural network (DNN) model by using the kinematics and kinetics data of runners with different weekly running mileages as input variables to classify and recognize the gait characteristics of runners with higher-mileage and low-mileage runners. (2) To evaluate the classifier performance of DNN classification models based on different input variables (separate kinematic inputs; separate kinetic inputs; kinematic and kinetic inputs together). (3) To identify the relevance of relevant variables and time points between higher-mileage and low-mileage runners by using LRP technology. (4) To explore LRP as a method for data reduction and explain the classification decision of the DNN classifier model based on the high relevant variables.

## Results

### Performance of deep neural network classification models

**Figure 1:**
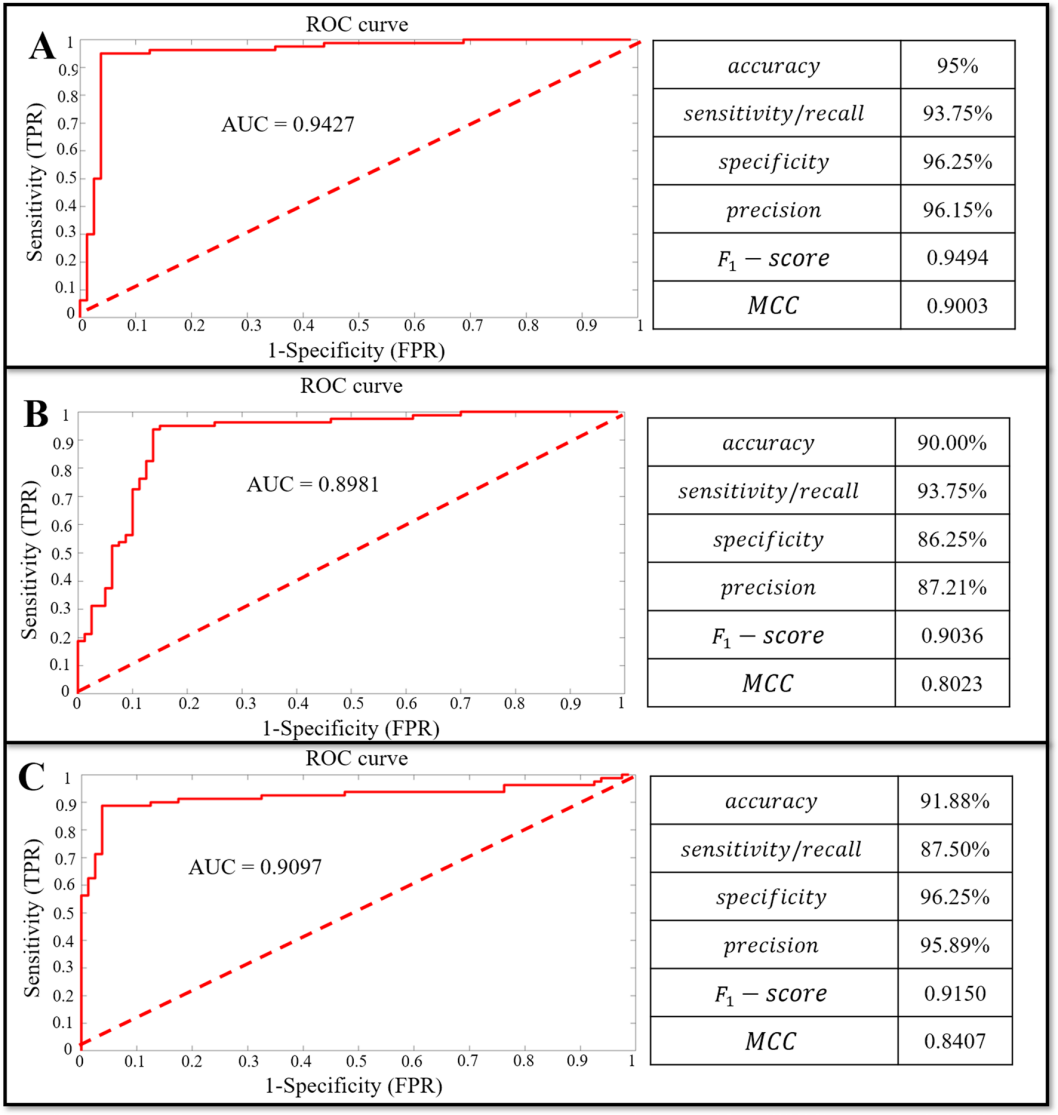
The classifier of the DNN models based on the matrices $M$, ${M}_{kinematics}$, ${M}_{kinetics}$. (**A**) The classifier of the DNN models based on the matrices $M$. (**B**) The classifier of the DNN models based on the matrices ${M}_{kinematics}$. (**C**) The classifier of the DNN models based on the matrices ${M}_{kinetics}$. ROC: Receiver Operating Characteristic; AUC: Area Under the ROC Curve; MCC: Matthews Correlation Coefficient; TPR: True Positive Rate; FPR: False Positive Rate.

For the matrices $M$, 75 TP, 5 FN, 77 TN and 3 FP were obtained by DNN classifier. For the matrices ${M}_{kinematics}$, 75 TP, 5 FN, 69 TN and 11 FP were obtained by DNN classifier. For the matrices ${M}_{kinetics}$, 70 TP, 10 FN, 77 TN and 3 FP were obtained by DNN classifier. All classification performance parameters are presented in Fig. 1. For the classifier of the DNN models based on the matrices $M$ (Fig. 1A), the model showed the higher accuracy rate (accuracy rate: 95%) than the matrices ${M}_{kinematics}$ (accuracy rate: 90.00%) and matrices ${M}_{kinetics}$ (accuracy rate: 91.88%). In general, the classifier of the DNN models based on the matrices $M$ presented a perfect accuracy rate, specificity rate, as well as precision rate compared to separate matrices ${M}_{kinematics}$ and ${M}_{kinetics}$. At the same time, the classifier of the DNN models based on the matrices $M$ showed the higher ${F}_{1}-score$ (0.9494) and $MCC$ (0.9003) than the matrices ${M}_{kinematics}$ and matrices ${M}_{kinetics}$ (Fig. 1C). Overall, the classifier performance based on the matrices $M$ achieved an ${F}_{1}-score$ and $MCC$ score of very strong relationships.

The ROC curves are showed in Fig. 1, the ROC curves of the classifier of the DNN models based on the matrices $M$ (Fig. 1A) presented a good classification performance during the overall area. However, the ROC curves based on the matrices ${M}_{kinematics}$ (Fig. 1B) show the worse classification performance during the about ${(0}_{FPR}{-0.1}_{FPR})*{(0.4}_{FPR}{-1}_{FPR})$ area, and the matrices ${M}_{kinetics}$ (Fig. 1C) show the worse classification performance during the about ${(0}_{FPR}{-0.7}_{FPR})*{(0.9}_{FPR}{-1}_{FPR})$ area. The classifier of the DNN models based on the matrices $M$ show the higher AUC (0.9427) than the matrices ${M}_{kinematics}$ (AUC: 0.8981) and matrices ${M}_{kinetics}$ (AUC: 0.9097). Overall, the classifier of the DNN models based on the matrices $M$ has a good performance from the perspective of overall indicators.

### Results of LPR

**Figure 2:**
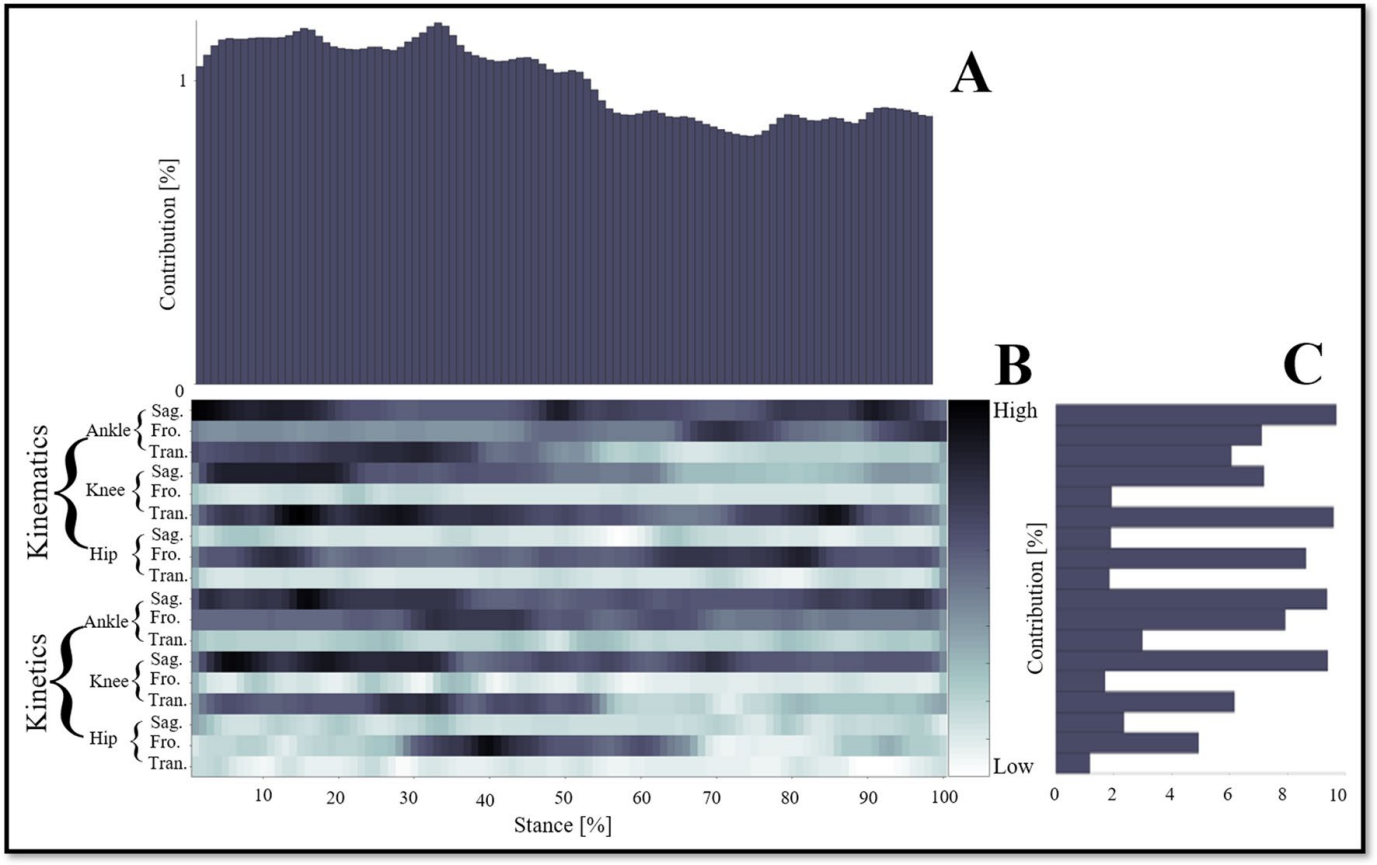
The LPR results in the average absolute relevance score of every variable in a stride pattern. (**A**) The relative contribution of variables during the overall stance phase (0%–100%). (**B**) The detailed distribution of relevance score during each joint (ankle, knee, hip) of each plane (sagittal, frontal, transverse) of kinematics (joint angle) and kinetics (joint moment). The darker colors mean high relevance variables, the lighter colors mean low relevance variables. The model relied more on darker color variables; the lighter colors variables had less relevance with correctly classified gait patterns. (**C**) The summed contribution of the relevance score of each joint (ankle, knee, hip) of each plane (sagittal, frontal, transverse) of kinematics (joint angle) and kinetics (joint moment) trajectories.

The relative contribution of variables during the overall stance phase are showed in Fig. 2A, the variables recorded at every 1% of the stance interval are related to successfully matching the stride pattern between the higher-mileage runners and lower-mileage runners. The contribution of variables during the 1%-47% stance phase (contribution: 52.54%) was higher than the contribution of variables during the 48%-100% stance phase (contribution: 47.46%) to the successful classification.

The summed contribution of the relevance score of each joint (ankle, knee, hip) of each plane (sagittal, frontal, transverse) of kinematics (joint angle) and kinetics (joint moment) trajectories are showed in Fig. 2C. The summed contribution rate of the relevance score of the ankle, knee, hip was 43.16%, 35.98%, 20.86%, respectively. The summed contribution rate of the relevance score of the sagittal, frontal, transverse was 39.90%, 32.24%, 27.86%, respectively. The most relevant trajectory variables were the ankle dorsiflexion-plantarflexion angle (9.69%), the knee internal–external rotation angle (9.59%), the ankle dorsiflexion-plantarflexion moment (9.37%), and the knee flexion–extension moment (9.39%). Secondly, the relevant trajectory variables were the knee flexion–extension angle (7.19%), the hip abduction–adduction angle (8.64%), and the ankle inversion-eversion moment (7.93%). However, there was little relevance score in the variables of knee abduction–adduction angle (1.93%), hip flexion–extension angle (1.90%), hip internal–external rotation angle (1.85%), ankle internal–external rotation moment (2.99%), knee abduction–adduction moment (1.70%), hip flexion–extension moment (2.36%), hip internal–external rotation moment (1.18%).

The detailed distribution of relevance score during each joint (ankle, knee, hip) of each plane (sagittal, frontal, transverse) of kinematics (joint angle) and kinetics (joint moment) are showed in Fig. 2B. There were revealing findings contributing to distribution of the variables on time points between the higher-mileage runners and lower-mileage runners during the overground running movement patterns.

**Figure 3:**
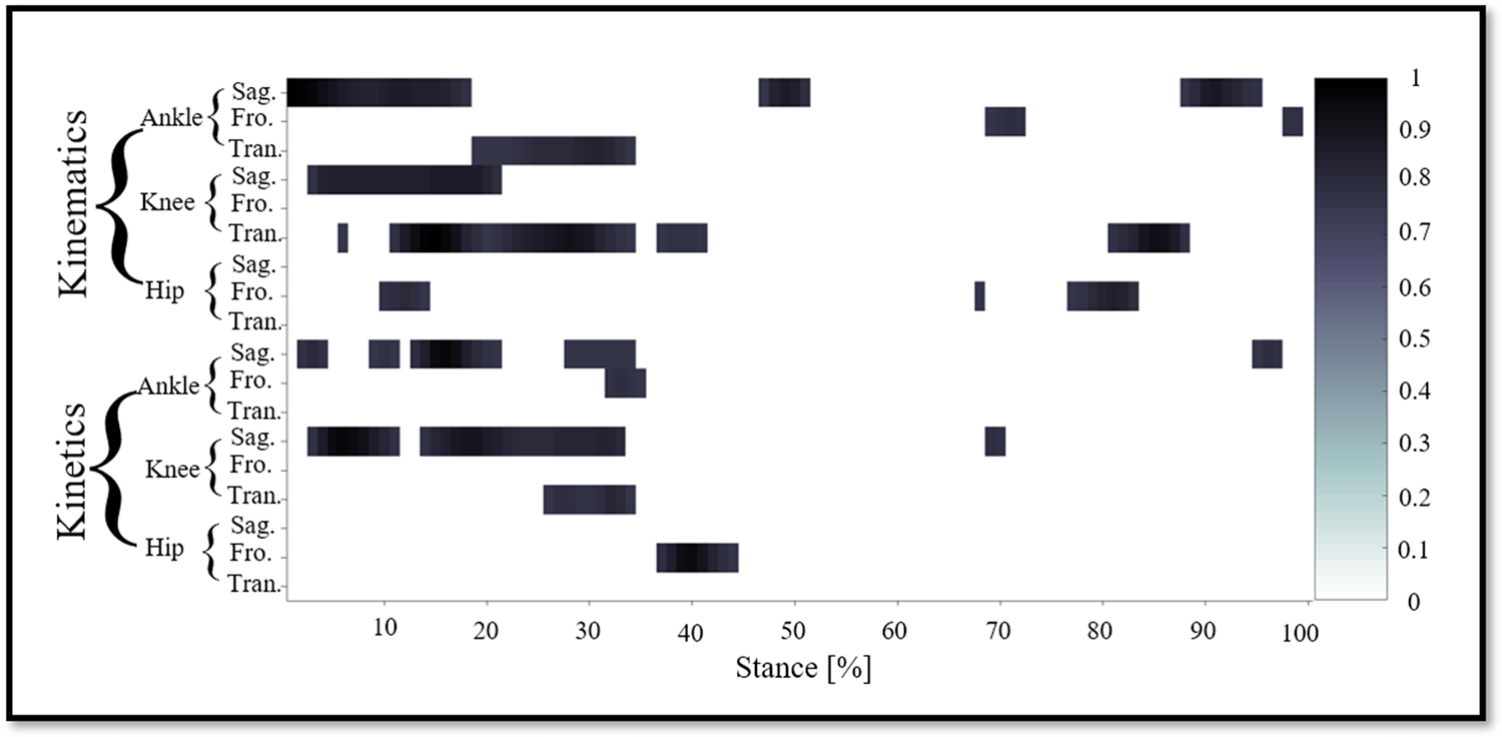
Notable highly relevant variable during each joint (ankle, knee, hip) of each plane (sagittal, frontal, transverse) of kinematics (joint angle) and kinetics (joint moment). The top 200 variables with the highest correlation relevance, all of them had a relevance score of over 0.7.

Notable highly relevant variables (the top 200 variables with the highest correlation relevance, all of them had a relevance score of over 0.7) during the stance are showed in Fig. 3. For the kinematics of the ankle, there was high relevance score in dorsiflexion-plantarflexion angle during the 1%–18%, 47%–51%, 88%–95% stance phase; in inversion-eversion angle during the 69%–72%, 98%–99% stance phase; in internal–external rotation angle during the 19%–34% stance phase. For the kinematics of the knee, there was high relevance score in flexion–extension angle during the 3%-21% stance phase; in internal–external rotation angle during the 6%, 11%–34%, 37%–41%, 81%–88% stance phase. For the kinematics of the hip, there was high relevance score in abduction–adduction angle during the 10%–14%, 68%, 77%–83% stance phase.

For the kinetics of the ankle, there was high relevance score in the dorsiflexion-plantarflexion moment during the 2%–4%, 9%–11%, 13%–21%, 28%–34%, 95%–97% stance phase; in the inversion-eversion moment during the 32%–35% stance phase. For the kinetics of the knee, there was high relevance score in the flexion–extension moment during the 3%–11%, 14%–33%, 69%–70% stance phase; in internal–external rotation moment during the 26%–34% stance phase. For the kinetics of the hip, there was high relevance score in the abduction–adduction moment during the 37%–44% stance phase.

## Discussion

This study aimed to investigate the differences in running gait patterns between higher-mileage runners and low-mileage runners. The objectives were to firstly train the DNN model by using the running gait kinematics (joint angle) and kinetics (joint moment) dataset as input variables to classify and recognize the gait characteristics of runners with higher-mileage and low-mileage runners. Secondly, to evaluate the classifier performance of DNN classification models based on different input variables (separate kinematic inputs; separate kinetic inputs; kinematic and kinetic inputs together). Finally, to use LRP to identify the relevance of relevant variables and time points between higher-mileage and low-mileage runners, and explain the classification decision of DNN classifier model based on those high relevant variables.

According to our research results, higher-mileage and low-mileage runners have discernable differences in gait characteristics, independently in relation to the perspective of kinematics or kinetics variables. When the classifier of the DNN models is only based on the kinematics as the input variables, the model shows good classification performance (Fig. 1A: accuracy rate is 90.00%). This supports previous findings of Clermont et al., who successfully classified higher- and low-mileage runners with 92.59% accuracy, showing that there are discernible differences in running gait kinematics between higher-mileage and low-mileage runners^12^. At the same time, when the classifier of the DNN models is only based on the kinetics as the input variables, the model accuracy rate is 91.88%, but when combining kinematics and kinetics as input variables, the model accuracy rate reaches 95%. In our study, the *F*_1_−*score* and *MCC* were used to evaluate the performance of the classifier, which can provide a good evaluation of the performance of the classifier^34,35^. In our results, the classifier of combining kinematics and kinetics as input variables obtained a higher *F*_1_−*score* (0.9494) and *MCC* (0.9003), as well as a higher AUC (0.9427). These results show that running gait kinetics data can increase the pattern recognition rate of gait characteristics between higher-mileage and low-mileage runners, at least in terms of classifier model performance. Therefore, the relevant research should consider the combination of kinematics and kinetics data sets rather than only simply kinematics when analyzing gait characteristics, if it is possible. It can provide more effective gait pattern information for the field of medical biomechanics. Of course, compared to only collecting kinematics data, both collecting kinematics and kinetics increase the difficulty of collection, especially in the absence of relevant equipment.

In the research of gait pattern recognition, it is often necessary to record a large amount of data in order to better recognize gait patterns^36^, which makes it difficult to complete an accurate interpretation of gait pattern recognition results with few variables as possible. In this study, the variables were imported into the DNN model for training, and then the relevance score of each variable's contribution to the gait pattern recognition results was obtained through LRP. The results of gait pattern recognition can be accurately interpreted by using highly correlated variables, which undoubtedly provides more important and effective information for gait pattern recognition. As shown in Fig. 2, not all variables contribute significantly to identifying the gait patterns of higher-mileage and low-mileage runners. The contribution of variables during the 1%–47% stance phase was higher than the contribution of variables during the 48%–100% stance phase to the successful recognize gait pattern (as shown in Fig. 2A). In other words, the early stage of the stance phase covers the interpretability of higher-mileage and low-mileage runners in gait pattern recognition. Horst et al. found that the most significant individual gait characteristics appeared in the early stage of the stance phase when they analyzed individual gait patterns in barefoot walking using LRP^10^. At the same time, Hoitz et al. found that the early stage of stance phase (1%–30%) has a more significant contribution to gait pattern recognition than the late stage of the stance phase^9^. The differences in foot strike patterns (from rearfoot strikes to forefoot strikes) are more readily observed in the early stages of stance^37^. These results seem to suggest that the early stages of stance may play a more important and meaningful role in identifying gait patterns. It also provides insights for other researchers who should focus on the early stages of stance when investigating gait patterns, at least for now the evidence suggests that early stages of stance contain more meaningful information about gait patterns.

In addition to showing a more significant contribution during the early stages of stance, the summed contribution of the relevance score of each joint of each plane of kinematics and kinetics trajectories are also inconsistent. As shown in Fig. 2C, our results show that the most relevant trajectory variables were the ankle dorsiflexion-plantarflexion angle, the knee internal–external rotation angle, the ankle dorsiflexion-plantarflexion moment, and the knee flexion–extension moment. The sagittal plane of the ankle and knee plays an important role in recognition gait patterns between high-milage and low-milage runners, which also confirms previous findings that the sagittal plane should be considered^11^. The hip appears to play a small role in identifying the gait patterns of higher-mileage and low-mileage runners, no matter from the perspective of kinematics or kinetics. However, when the top 200 variables with the highest correlation relevance score (as shown in Fig. 3, all of them had a relevance score of over 0.7) were extracted^9^, the high relevance score was shown in the abduction–adduction angle (moment) during the 10%–14%, 68%, 77%–83% (37%–44%) stance phase. Previous studies have shown that high-mileage runners exhibit larger hip adduction and have a higher risk of hip injury compared to low-mileage runners^7,11^. Therefore, it is permissible to use the gait characteristic of the hip frontal plane to identify gait patterns in higher-mileage and low-mileage runners, which can provide more information about injuries and individual characteristics. At the same time, the ankle and knee provide considerable information to recognize gait features, especially in the sagittal and transverse planes. It also suggests that runners adjust their gait patterns during the running gait stance phase, leading to more flexion of the knee and more valgus of the foot^12,38^. Therefore, the high-mileage runners show higher rates of hip and hamstring injuries and low-mileage runners show higher rates of knee injuries may be due to their different gait patterns.

In general, LRP completes a feasible interpretation of the predicted results of the model, thus providing more interesting insights and more effective information for analyzing gait patterns. The relevance score results of LRP output enable machine learning algorithms (such as artificial neural networks) to predict and analyze multiple variables of the gait cycle from different time points. Compared with traditional gait analysis methods (based on a single pre-selected variable), machine learning algorithms in the field of medical biomechanics seem to be better able to correlate human movement with related injuries and diseases in multiple dimensions^16,39^. At the same time, the explainable relevance score results of gait pattern recognition show that the variables related to a particular gait pattern recognition are not confined to a single gait feature, nor are they evenly distributed across all gait features. In summary, the results of LRP demonstrate its applicability to the understanding and interpretation of machine learning prediction results in clinical (biomechanical) gait analysis. In other words, the application of machine learning in gait analysis combined with LRP is well worth considering by researchers, which also provides some references for future clinical (biomechanical) analysis and diagnostic research.

The current study has some limitations. First of all, only male runners were included in this study, so the results of this study apply only to male runners. In the future, female runners can be combined to explore the differences in gait patterns among different mileage runners. Secondly, the current study used uniform runners' running speeds (3.3 m/s ± 10%) to minimize the biomechanical differences due to different running speeds^40^. Because of the differences in training levels and running habits between high-mileage and low-mileage runners, there may be a small number of runners not showing the most realistic gait pattern. In general, however, the subjects were given enough time to familiarize themselves to the uniform speed prior to formal experimental data collection, which compensated for any possible errors outlined.

## Conclusion

Considering the combination of kinematics and kinetics data sets rather than only simply kinematics when analyzing gait characteristics can increase the pattern recognition rate of gait characteristics between higher-mileage and low-mileage runners, as well as providing more effective and efficient gait pattern information. The ankle and knee provide considerable information that can help recognize gait features, especially in the sagittal and transverse planes. This may be the reason why high-mileage and low-mileage runners have different injury patterns due to their different gait patterns. The early stages of the stance are also very important in the term of gait pattern recognition because it contains more effective information about gait patterns. LRP completes a feasible interpretation of the predicted results of the model, thus providing more interesting insights and more effective information for analyzing gait patterns. Thus, researchers should consider combining LRP when they apply machine learning in gait analysis.

## Methods

### Participants

This study recruited 80 male healthy runners: 40 higher-mileage runners (age: 35.51 ± 10.32 y, height: 172.30 ± 8.13 cm, body mass: 65.33 ± 7.46 kg, running experience: 8.56 ± 7.74, weekly mileage: 44.31 ± 13.67 km), 40 lower-mileage runners (age: 33.90 ± 9.74 y, height: 173.40 ± 6.96 cm, body mass: 68.58 ± 8.20 kg, running experience: 4.71 ± 3.19, weekly mileage: 15.28 ± 5.30 km). The criteria for inclusion were no serious lower extremity musculoskeletal injury, no history of major lower extremity surgery, or any other injury factors that might interfere with the study in the previous 6 months. According to previous studies^11,12^, “lower-mileage” runners were defined as those who self-reported running less than 25 km per week, while “higher-mileage” runners were defined as those who ran more than 32 km per week. Participants were informed of the purpose, requirements, and procedures of the experiment. This study was performed in accordance with the Declaration of Helsinki, the study protocol was approved (Approval Number: RAGH20210326) by the Ethics Committee of Ningbo University, and the written informed consent was provided and signed by all subjects.

### Experimental protocol and procedures

The experiment was conducted in the biomechanics laboratory at the Research Academy of Grand Health, Ningbo University. Three-dimensional lower limb joint kinematics data were collected at 200 Hz using a Vicon (Vicon Metrics Ltd., United Kingdom) motion capture system (eight Infrared cameras). In an identical time frame, the ground reaction force (GRF) data were synchronously collected using a 1000 Hz in-ground AMTI force plate (AMTI, Watertown, United States). Vicon motion capture system and AMTI force plate are connected through Vicon Nexus 1.8.6 software to achieve the synchronous collection. This study selected the right leg as the analytical limb, so the 12.5 mm diameter standard reflective marker was attached to the pelvis and right lower limb^25^: right anterior superior iliac spine, left anterior superior iliac spine, right posterior superior iliac spine, left posterior superior iliac spine, right medial condyle, right lateral condyle, right medial malleolus, right lateral malleolus, right first metatarsal head, right fifth metatarsal head, right distal interphalangeal joint of the second toe. At the same time, three tracking clusters were labeled on the right middle and lateral thigh, right middle and lateral shank, right heel. A stadiometer and a calibrated scale were used to measure the subject’s body mass and height respectively.

All subjects were asked to wear leggings and tights and uniform standard running shoes (Anta Flashedge, China). All runners were heel strikers. Prior to the formal experiment, subjects warmed up by jogging for 10 min in the laboratory environment at a self-selected speed. Following warm up, they then familiarized themselves with the experiment process and conducted preliminary experimental data collection. The infrared timers were placed on either side of the 20-m track to measure the participants' running speed (specific location: 4-m behind/in front of the force plate). The subjects were asked to run naturally across the track at a speed of 3.3 ± 10% meters per second and land with their right foot on the force plate in a natural unconsciousness way^26^. The test was considered valid when the subject was observed and measured to run at the correct speed and in a natural manner. A total of 10 recordings of valid data were collected for each subject.

## Data collection and processing

Based on the study of Xu et al.^27^, the initial contact force point was determined as the vertical GRF greater than 10 N. The stance phase was defined as the force plate from the initial contact force point to the right lower limb leaving the force plate (force value to zero). The whole data set was preprocessed using Vicon Nexus 1.8.6 software. Firstly, the data of the reflective marker trajectory coordinates and the GRF data are exported from Vicon Nexus into C3D format file, and then the C3D format file is imported into Visual 3-D software (version 6.7.3, C-Motion Inc., Germantown, United States) for modeling and further processing. According to Winter’s study in relation to the filter selected frequency, the most appropriate signal-to-noise ratio was selected by carrying out residual analysis of the data of subsets^28^. Finally, fourth-order zero-phase lag Butterworth low-pass filters were selected to filter the data (Filter frequency, kinematics data: 10 Hz, kinetic data: 20 Hz). The pelvis model was developed according to the CODA model, and the hip joint center location was defined by regression Eqs. ^29^. The right hip joint center (RHJC) according to Eq. (1) and left hip joint center (LHJC) according to Eq. (2) was identified by the anterior superior iliac spine (ASIS):

1 $$ RHJC = \left( {0.36*ASIS_{Distance} , - 0.19*ASIS_{Distance} , - 0.3*ASIS\_Distance} \right) $$

2 $$ LHJC = \left( { - 0.36*ASIS_{Distance} , - 0.19*ASIS_{Distance} , - 0.3*ASIS\_Distance} \right) $$

The center position of each segment was determined by the coordinates of the reflective markers, and then the joint angles of each segment were calculated. Finally, the joint kinetics (joint moment) was calculated by the inverse kinetics algorithm in Visual 3-D software. All joint kinematics and joint kinetics data were then imported into MATLAB R2019a (Visual R2019a, MathWorks, United States) to process further. For each joint (ankle, knee, hip) of each plane (sagittal, frontal, transverse) of kinematics (joint angle) and kinetics (joint moment) data, all were extracted to expand into 100 data point curves by custom MATLAB script. Finally, two matrices can be obtained:

$$ M_{kinematics} = 800 \left( {80\, subjects*10\, trials} \right)*900 \left( {3\, joint*3\, plane*100\, data\, points} \right) $$

$$ M_{kinetics} = 800 \left( {80\, subjects*10\, trials} \right)*900 \left( {3\, joint*3 plane*100\, data\, points} \right) $$

## Data analysis

**Figure 4:**
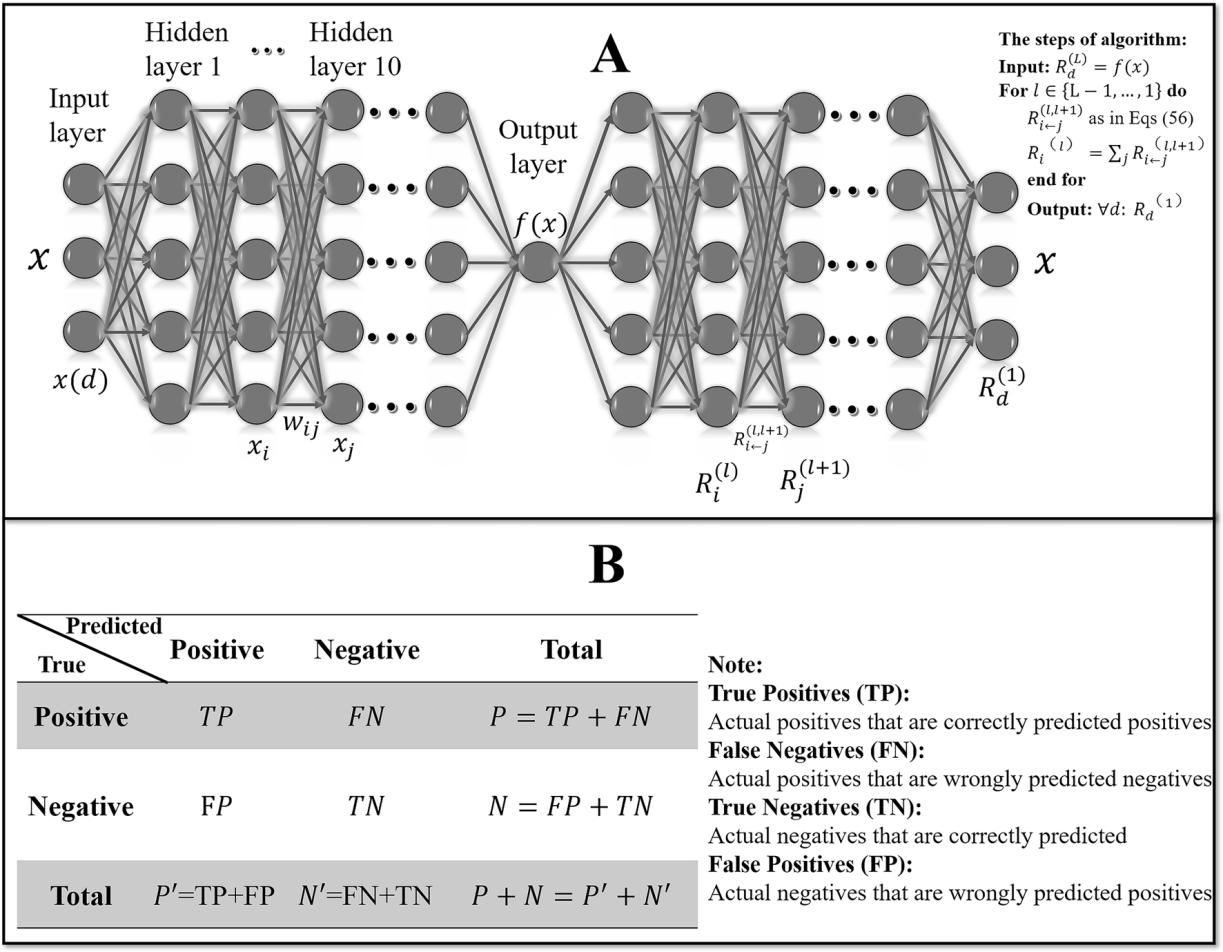
(**A**) A description of the neurons and weight connections of the DNN by the interpretation of the different variables and indices from multilayers. Left is the process of establishing $f\left(x\right)$ by forward pass of DNN. Right is the process of calculating relevance score ${R}_{d}^{(1)}$ by LRP back pass. On the upper right side is the algorithm summary about the complete LRP procedure for DNN. (**B**) A description of the confusion matrix of binary classifier.

Neural networks are widely parallel networks of adaptive simple units whose organization can simulate the interactions of biological nervous systems to real-world objects^30^. Neural networks with more than two hidden layers are defined as deep neural networks, and deep neural network (DNN) is generally considered to improve the accuracy of the whole model^31^. The application of the DNN model in this study was mainly biased to improve the accuracy of the model, so a DNN model with ten hidden layers was designed under the condition of repeated model training and adjustment according to the actual data. The matrices $M_{kinematics}$, $M_{kinetics}$, and $M = M_{kinematics} + M_{kinetics}$ was conducted using Layer-wise Relevance Propagation (LRP) respectively. Firstly, a deep neural network (DNN) was established that included one input layer, ten hidden layers, and one output layers, and the per layer nodes were determined by the input data shape^32^. Therefore, for the dataset $M_{kinematics}$ and $M_{kinetics}$, the nodes of the input layer, hidden layers, and output layer were 900, 1800, and 2. For the dataset $M$, the nodes of the input layer, hidden layers, and output layer were 1800, 3600, and 2. As shown in Fig. 4A, the layers of the neural network are fully connected, which means the neuron of the $n$-th layers must be connected to the neuron of the $\left( {n + 1} \right)$-t h layer. A linear relation function and an activation function were used to calculate the new values between layers, and the linear relationship function of the model constructed in this study was

3 $$ z = \mathop \sum \limits_{i = 1}^{m} w_{i} x_{i} + b $$

The $w_{i}$ is the connection weight of the $i$-th neuron, and the $x_{i}$ is the input from the $i$-th neuron. The hidden layer activation function was used the hyperbolic tangent function

4 $$ gx = \frac{{e^{x} - e^{ - x} }}{{e^{x} + e^{ - x} }} $$

The batch size was set 25, and the epoch limit was set 3000. At the same time, the data of the higher-mileage runner was set at positive class, and the data of the lower-mileage runner was set to negative class. Before the data training, the 10 data sets of successful trials for each subject were taken as a whole, and then randomly extracted the data sets of 32 higher-mileage and 32 lower-mileage runners as training sets (a total of 640 sample data sets), the remaining data sets of 8 higher-mileage and 8 lower-mileage runners as test sets (a total of 160 sample data sets). Following DNN training, the relevance score was calculated by the LRP, and the performance of the classifier was evaluated by the accuracy achieved and other parameters.

### Layer-wise relevance propagation

Layer-wise Relevance Propagation (LRP) is technology used to identify important relevance through backward propagation in neural networks. Backward propagation is a conservative relevance redistribution process in which the neurons that contribute the most to the upper layer receive the most relevance from the upper layer. In general, LRP aims to narrow the gap between the classification and interpretability of multi-layer neural networks on nonlinear cores^22,23^.

The overall idea is to understand the contribution of a single feature of dataset $x$ to the prediction $f\left( x \right)$ made by the classifier $f$ in pattern recognition and classification tasks. That is, the positive or negative contribution of each feature to the classification result for dataset $x$ can be calculated, and the degree of such contribution can be accurately measured to a certain extent (The contribution of each input feature $x\left( d \right)$ to a particular prediction $f\left( x \right)$. In the setting of the classifier is a mapping $f$: $R^{v} \to R^{1}$, $f\left( x \right) > 0$ indicates the existence of a learning structure. The constraint of classification is to find the differential contribution relative to the most uncertain state of the classification, which is then represented by the root point $f\left( {x_{0} } \right) = 0$. By factoring the prediction $f\left( x \right)$ into the sum of the individual input feature $x\left( d \right)$:

5 $$ f\left( x \right) = \mathop \sum \limits_{d = 1}^{V} R_{d} $$

In the classifier, whether for nonlinear support vector machines or neural networks, the first layer is the input features, and the last layer is the predicted output of the classifier. Meanwhile, each layer is part of the features extracted from the dataset $x$ after running the classification algorithm. The $l$-th layer is modeled as a vector $z = \left( {z_{d}^{l} } \right)_{d = 1}^{V\left( l \right)}$ with dimensionality $V\left( l \right)$. LRP has a relevance score $R_{d}^{{\left( {l + 1} \right)}}$ for each dimension $z_{d}^{{\left( {l + 1} \right)}}$ of vector $z$ at layer $l + 1$. A relevance score $R_{d}^{\left( l \right)}$ is found in each dimension $z_{d}^{l}$ of vector $z$ near the next layer $l$ of the input layer, as shown in the following formula:

6 $$ f\left( x \right) = \cdots = \mathop \sum \limits_{d \in l + 1} R_{d}^{l + 1} = \mathop \sum \limits_{d \in l} R_{d}^{l} = \cdots = \mathop \sum \limits_{d} R_{d}^{1} $$

The inter-hierarchical relevance is represented by the message $R_{i \leftarrow j}^{l, l + 1}$ between neuron $i$ and $j$, and these messages can be sent along with each connection. As shown in Fig. 4A, the output $f\left( x \right)$ is then passed from one neuron to the next by backward propagation. The relevance of neurons is defined as the sum of incoming messages, then the sum runs over the sinks at layer $l + 1$ for a fixed neuron $i$ at a layer $l$.

7 $$ R_{j}^{\left( l \right)} = \mathop \sum \limits_{k: i\, is\, input\, for\, neuron\, k} R_{i \leftarrow k}^{{\left( {l, l + 1} \right)}} \left( 7 \right) $$

The Input of the next neuron in the direction defined during classification, then the sum runs over the sources at layer $l$ for a fixed neuron $k$ at layer $l + 1$. In general, this can be expressed as:

8 $$ R_{k}^{{\left( {l + 1} \right)}} = \mathop \sum \limits_{i: i\, is \,input\, for\, neuron \,k} R_{i \leftarrow k}^{{\left( {l, l + 1} \right)}} $$

The relevance of each layer is calculated by backward propagation: the relevance $R_{i}^{\left( l \right)}$ is expressed as a function of the upper relevance $R_{j}^{{\left( {l + 1} \right)}}$, and back propagates the relevance until the input feature is reached. As shown in Fig. 4A, through the relevance of the neuron $R_{j}^{{\left( {l + 1} \right)}}$ to the classification decision $f\left( x \right)$, the relevance is then decomposed according to the message $R_{i \leftarrow j}$ sent to the upper layer of neurons. Holding the conservation property:

9 $$ \mathop \sum \limits_{i} R_{i \leftarrow j}^{{\left( {l, l + 1} \right)}} = R_{j}^{{\left( {l + 1} \right)}} $$

For the linear network $f\left( x \right) = \mathop \sum \limits_{i} z_{ij}$, the relevance is $R_{j} = f\left( x \right)$, and the decomposition directly by $R_{i \leftarrow j} = z_{ij}$. Through hyperbolic tangent function and rectification function two monotone increasing functions, the pre-activation function ${z}_{ij}$ provides a reasonable way to measure the relative contribution of $x_{i}$ to $R_{j}$ for each neuron. Based on the proportion of local pre-activation and global pre-activation, the selection of association decomposition is obtained:

10 $$ R_{i \leftarrow j}^{l, l + 1} = \frac{{z_{ij} }}{{z_{j} }}*R_{j}^{l + 1} $$

The relevance $R_{i \leftarrow j}$ are shown in:

11 $$ \mathop \sum \limits_{i} R_{i \leftarrow j}^{{\left( {l, l + 1} \right)}} = R_{j}^{{\left( {l + 1} \right)}} *\left( {1 - \frac{{b_{j} }}{{z_{j} }}} \right) $$

Multiplier accounts represent the relevance absorbed by the bias term, and the residual bias correlations can be reassigned to each neuron $x_{i}$. According to the determined rule (Eq. 10), through adding up the correlations of all neurons in the upper layer $i$ (combined Eqs. (7) and (8)), the overall relevance of all neurons in the next layer $j$ can be obtained:

12 $$ R_{i}^{\left( l \right)} = \mathop \sum \limits_{j} R_{i \leftarrow j}^{{\left( {l, l + 1} \right)}} $$

The relevance propagates from one layer to another until it reaches the input feature $x\left( d \right)$, where the relevance $R_{d}^{\left( 1 \right)}$ provides the hierarchical eigen-decomposition required for the decision $f\left( x \right)$. The upper right side of Fig. 4A summarized the algorithm of the complete LRP procedure for DNN. More details can be found by referring to Lapuschkin et al^22^. All algorithms were run in MATLAB R2019a (Natick, Massachusetts: The MathWorks Inc.), through self-written scripts according to the layer-wise relevance propagation toolbox^33^.

The relevance of correctly classified gait patterns was extracted by defining logical variables, and then a relevance score was assigned to each input variable. LRP determines the correlation between each variable and the predicted results of the model, and normalizes the LRP-derived association patterns to their respective maximum values for comparison. After then, the average of all relevant patterns was determined and the error was rectified. The rectified average was smoothed, whereby the present point was weighted with 50%, and the previous and following points were weighted with 25%. For the smoothing process, the weighted values were set such that their total equaled 1 and a repetition of the procedure would approximate a Gaussian filter. Each of these steps was performed three times to get the desired result. Finally, the smoothed correlation pattern was rescaled from 0 (no correlation) to 1 (the highest correlation)^9^. Since the input variables are collected in the time domain, and the adjacent values are interdependent, the fluctuation of the relevance score can be reduced by smoothing. To explore the influence of different variables on the accuracy of model classification, all variables were sorted according to the correlation between variables, and then the top 200 variables with the highest relevance scores were selected to explain and analyze the gait pattern.

### Evaluate the performance of the classifier

Combine the results of the classification model into a $2*2$ table called confusion matrix $$ {\varvec{m}} = \left( {\begin{array}{*{20}c} {TP} & {FN} \\ {FP} & {TN} \\ \end{array} } \right)$$ (more details are shown in Fig. 4B) which fully describes the results of the classification task^34^. Then, the following indicators were calculated to evaluate the performance of the classifier.

1. The accuracy of a classifier on a given set of tests is the percentage of tuples that are correctly classified by the classifier:
  $$ accuracy = \frac{TP + TN}{{P + N}} $$
2. The sensitivity (also called recall) is the true positive cases recognition rate, which means the percentage of positive tuples correctly identified:
  $$ sensitivity/recall = \frac{TP}{{TP + FN}} $$
3. The specificity is the true positive cases recognition rate, which means the percentage of negative tuples correctly identified:
  $$ specificity = \frac{TN}{{FP + TN}} $$
4. The precision is a measure of accuracy, which means the percentage of tuples marked as positive that are actually positive:
  $$ precision = \frac{TP}{{TP + FP}} $$
5. $F_{1} - score$ is the harmonic average of accuracy and recall rate, which means the recall rate is weighted once as much as the precision:
  $$ F_{1} - score = \frac{2*precision*recall}{{precision + recall}} $$
6. Receiver Operating Characteristic (ROC) curves is a useful visual tool for comparing classifier models, which can provide objective and neutral advice regardless of cost/benefit when making decisions. The ROC curve shows the tradeoff between the true positive rate (TPR) and the false positive rate (FPR) for the classifier model. The increase in TPR comes at the expense of the increase in FPR:
  $$ TPR = \frac{TP}{{TP + FN}} $$
  $$ FPR = \frac{FP}{{FP + TN}} $$
  The Y-axis of the ROC curve represents TPR and the X-axis represents FPR, and the area under the ROC curve ($AUC$) is a measure of model accuracy:
  $$ AUC = \frac{{\left( {TPR - FPR + 1} \right)}}{2} $$
7. Matthew’s correlation coefficient (MCC) is a contingency matrix method^34^. MCC can be used to calculate the Pearson product-moment correlation coefficient ^35^ between the actual value and the predicted value:
  $$ MCC = \frac{TP*TN - FP*FN}{{\sqrt {\left( {TP + FP} \right)*\left( {TP + FN} \right)*\left( {TN + FP} \right)*\left( {TN + FN} \right)} }} $$
